# Supplementary figures and images for: Assessing attention towards plants: Development and first steps to the validation of the Hidden Object Picture Instrument (HOPI)
Source: PLoS One. 2026 May 21;21(5):e0349383. doi: 10.1371/journal.pone.0349383 (PMC13193508; doi:10.1371/journal.pone.0349383)

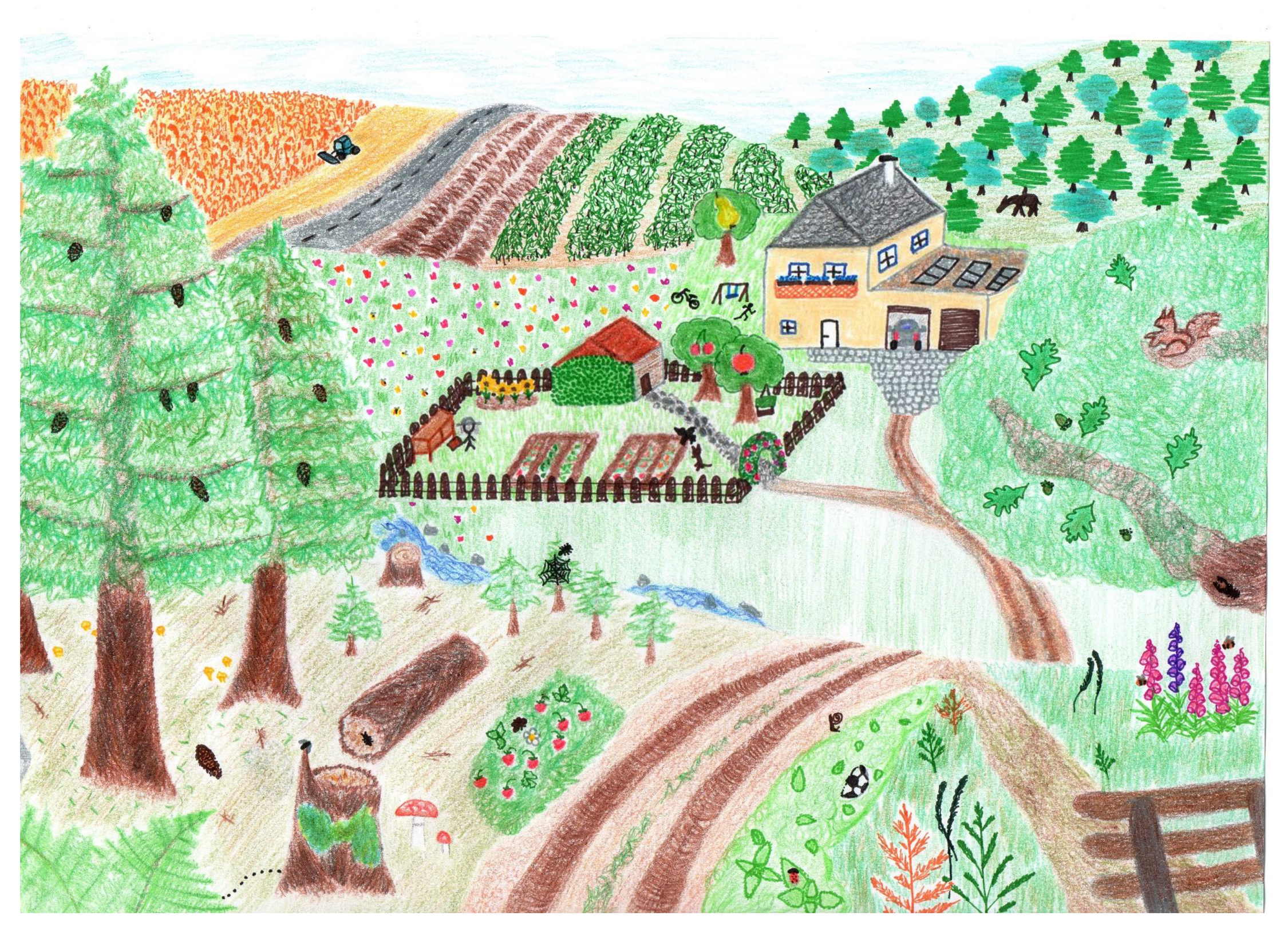

Supplement: S1 Fig — (TIF) [file pone.0349383.s001.tif]

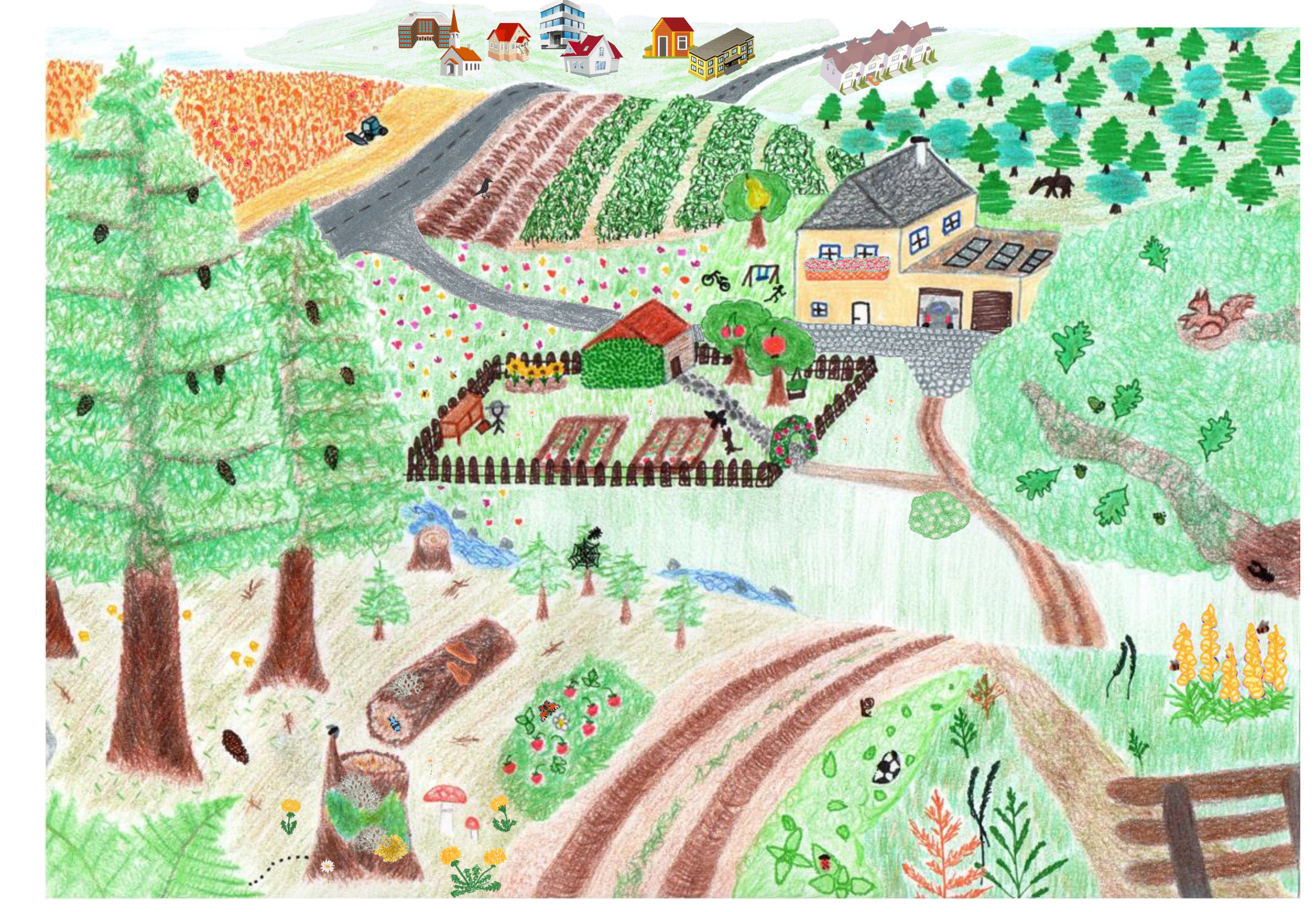

Supplement: S2 Fig — (TIF) [file pone.0349383.s002.tif]
