# Supplementary material for: Assessing attention towards plants: Development and first steps to the validation of the Hidden Object Picture Instrument (HOPI)
Source: PLoS One. 2026 May 21;21(5):e0349383. doi: 10.1371/journal.pone.0349383 (PMC13193508; doi:10.1371/journal.pone.0349383)
Supplement: S1 File — List of all 46 species depicted in the hidden object picture. (DOCX) [file pone.0349383.s003.docx]

**List of all 46 species depicted in the hidden object picture**

Plants:

- Apple tree – *Malus domestica*
- Beech – *Fagus sylvatica*
- Beetroot – *Beta vulgaris*
- Carrots – *Daucus carota* subsp. *sativus*
- Cherry Tree – *Prunus avium*
- Corn – *Zea mays*
- Daisies – *Bellis perennis*
- Dandelion – *Taraxacum officinale*
- Elderberry – *Sambucus nigra*
- Foxglove – *Digitalis lutea*
- Golden Oatgrass – *Trisetum flavescens*
- Grass – *Poa nemoralis*
- Ivy – *Hedera helix*
- Lady fern – *Athyrium filix-femina*
- Lettuce – *Lactuca sativa*
- Oak – *Quercus robur*
- Pear tree – *Pyrus communis*
- Peas – *Pisum sativum*
- Pelargonium – *Pelargonium peltatum*
- Pepper – *Capsicum annuum*
- Poppy flowers – *Papaver rhoeas*
- Red clover – *Trifolium pratense*
- Roses – *Rosa* spp.
- Spruce – *Picea abies*
- Stinging nettle – *Urtica dioica*
- Sunflower – *Helianthus annuus*
- Tomato – *Solanum lycopersicum*
- Wheat – *Triticum aestivum*
- Wild strawberries – *Fragaria vesca*
- Wood sedge – *Carex sylvatica*

Animals:

- Alpine longhorn beetle – *Rosalia alpina*
- Basket spider – *Linyphia triangularis*
- Bumblebees – *Bombus* sp.
- Great tit – *Parus major*
- Ladybird – *Coccinella septempunctata*
- Roe deer – *Capreolus capreolus*
- Peacock butterfly – *Aglais io*
- Red wood ant – *Formica rufa*
- Edible snail – *Helix pomatia*
- Fly – *Musca domestica*
- Stag beetle – *Lucanus cervus*
- Squirrel – *Sciurus vulgaris*
- Crow – *Corvus corone corone*

Fungi:

- Chanterelle – *Cantharellus cibarius*
- Fly agaric – *Amanita muscaria*
- Tree sponge – *Fomes fomentarius*
