# Supplementary material for: Assessing attention towards plants: Development and first steps to the validation of the Hidden Object Picture Instrument (HOPI)
Source: PLoS One. 2026 May 21;21(5):e0349383. doi: 10.1371/journal.pone.0349383 (PMC13193508; doi:10.1371/journal.pone.0349383)
Supplement: S2 File — Text of the task for the HOPI. (PDF) [file pone.0349383.s004.pdf]

Dimension: Attention

Take your time and take a close look at the hidden object picture! After 30 seconds, a ‘Next’ button will appear. Click on it when you are ready to proceed. Otherwise, you will be automatically redirected after 60 seconds.

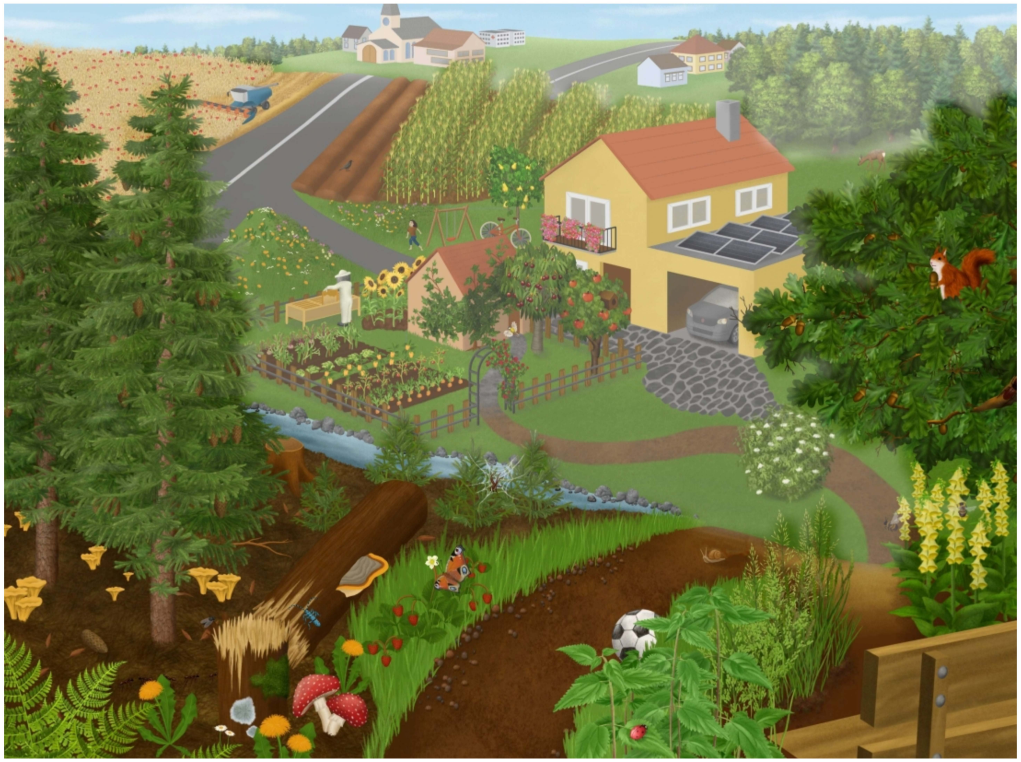

What did you see in the picture? Write down everything you can remember (you have space for a maximum of 25 words)!

When you have written an answer in a field and pressed the ENTER key, a new field automatically appears.
