# Supplementary material for: Assessing attention towards plants: Development and first steps to the validation of the Hidden Object Picture Instrument (HOPI)
Source: PLoS One. 2026 May 21;21(5):e0349383. doi: 10.1371/journal.pone.0349383 (PMC13193508; doi:10.1371/journal.pone.0349383)
Supplement: S3 File — Species identification questionnaire for the validation of the HOPI (English translation). (PDF) [file pone.0349383.s005.pdf]

How old are you?

Enter your age as a number!

What is your gender?

- male
- Male
- diverse

Which class are you in? Indicate which class you are in!

Instructions

You will now see 23 pictures of plants, animals and fungi. Please write the name of each species shown in the box below the picture. If you do not recognise the species shown, please leave the box blank.

7 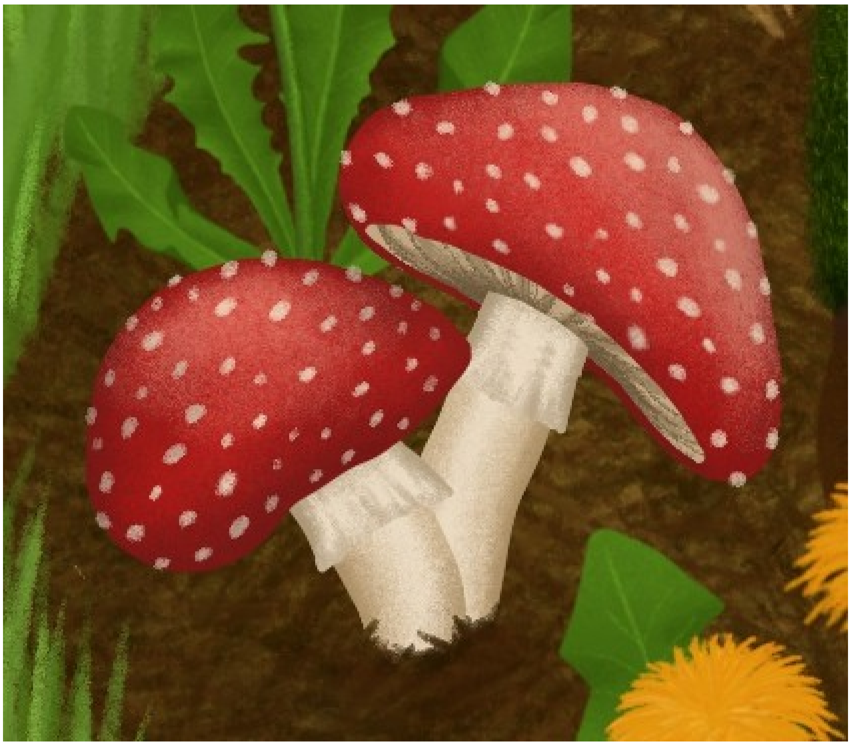

Please name the fungus shown (if you know it).

Enter the name in the field.

8. 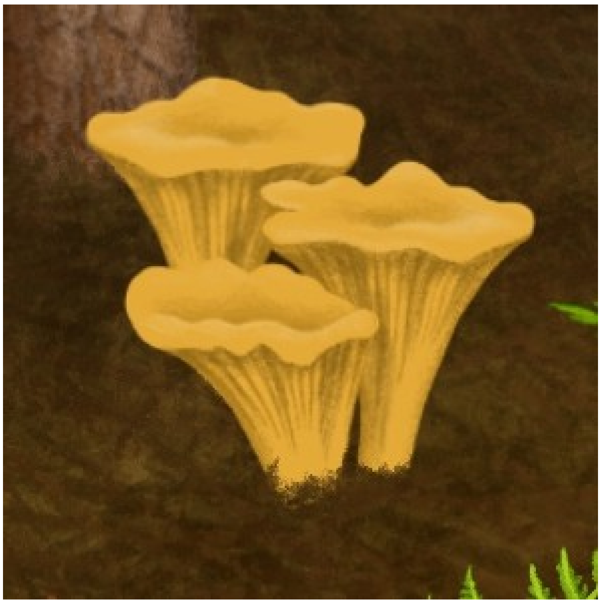

Please name the mushroom shown (if you know it).

Enter the name in the field.

9. 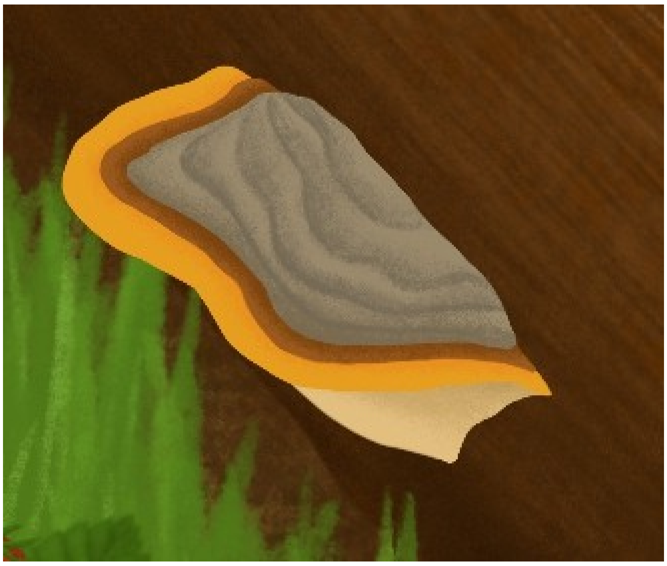

Please name the mushroom shown (if you know it).

Enter the name in the field.

10. 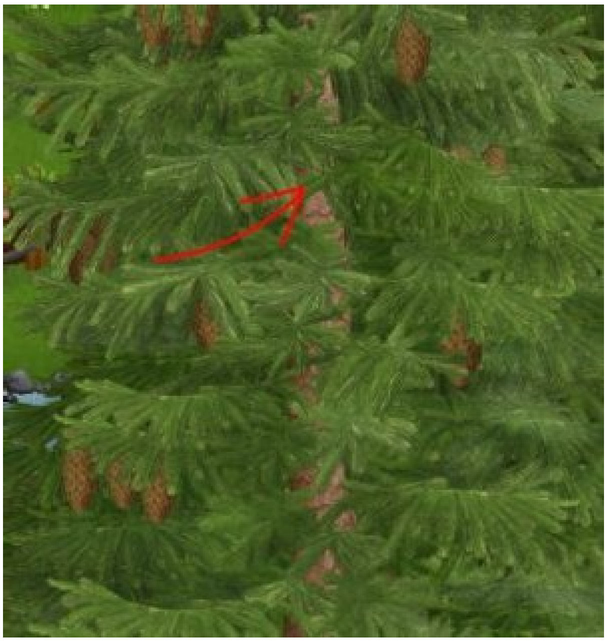

Please name the plant shown (if you know it).

Enter the name in the field.

11. 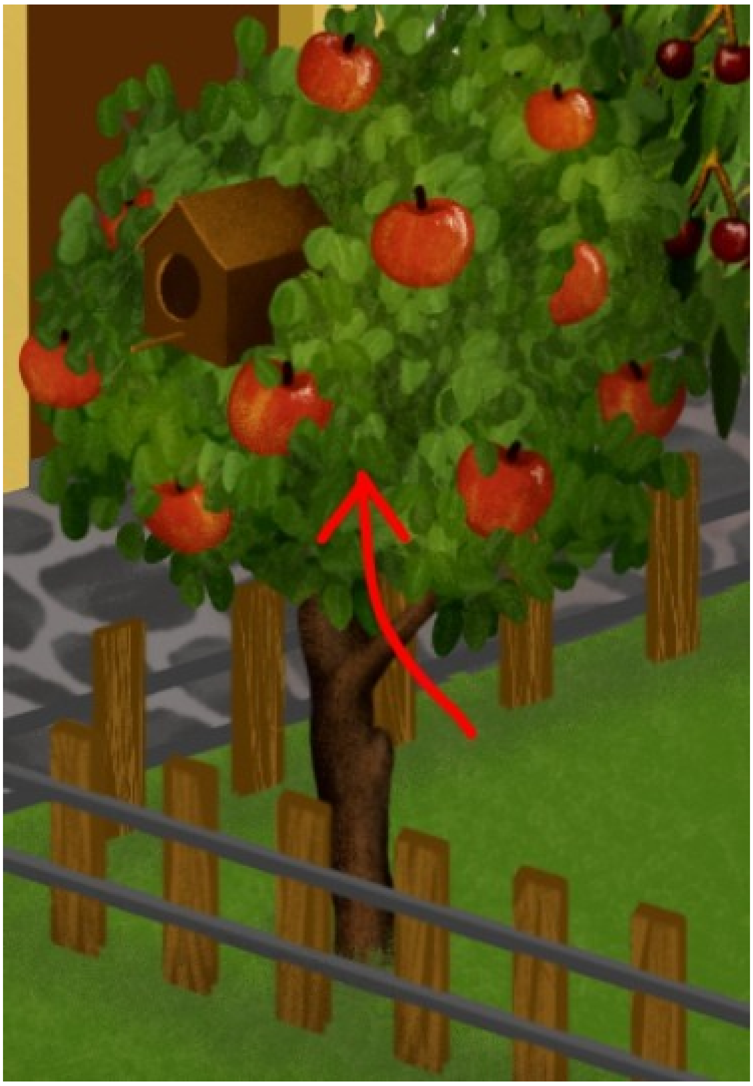

Please name the plant shown (if you know it).

Enter the name in the field.

12. 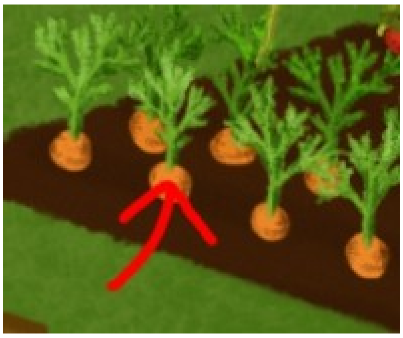

Please name the plant shown (if you know it).

Enter the name in the field.

13. 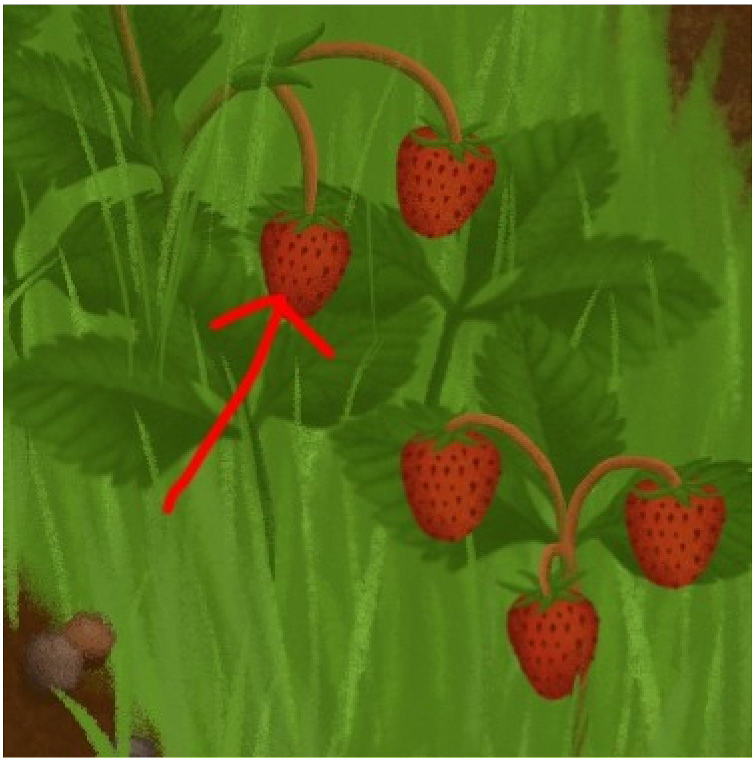

Please name the plant shown (if you know it).

Enter the name in the field.

14. 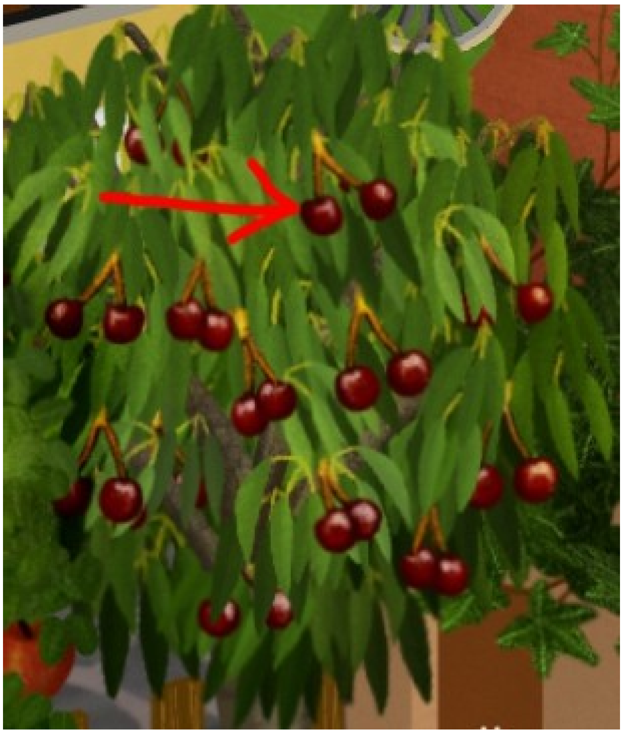

Please name the plant shown (if you know it).

Enter the name in the field.

15. 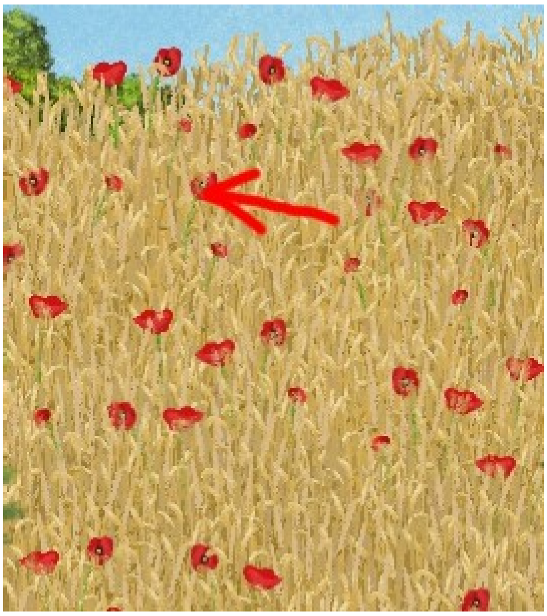

Please name the plant shown (if you know it).

Enter the name in the field.

16. 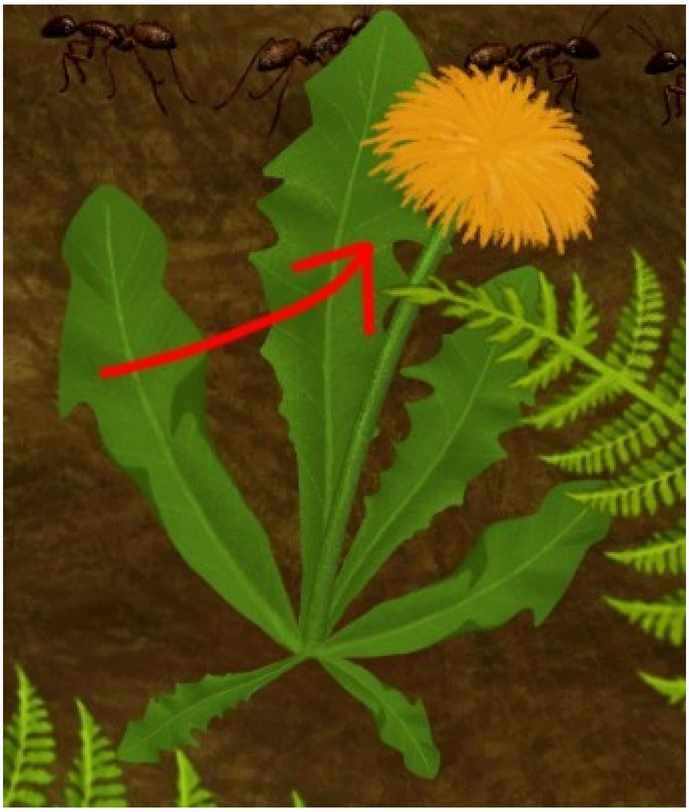

Please name the plant shown (if you know it).

Enter the name in the field.

17. 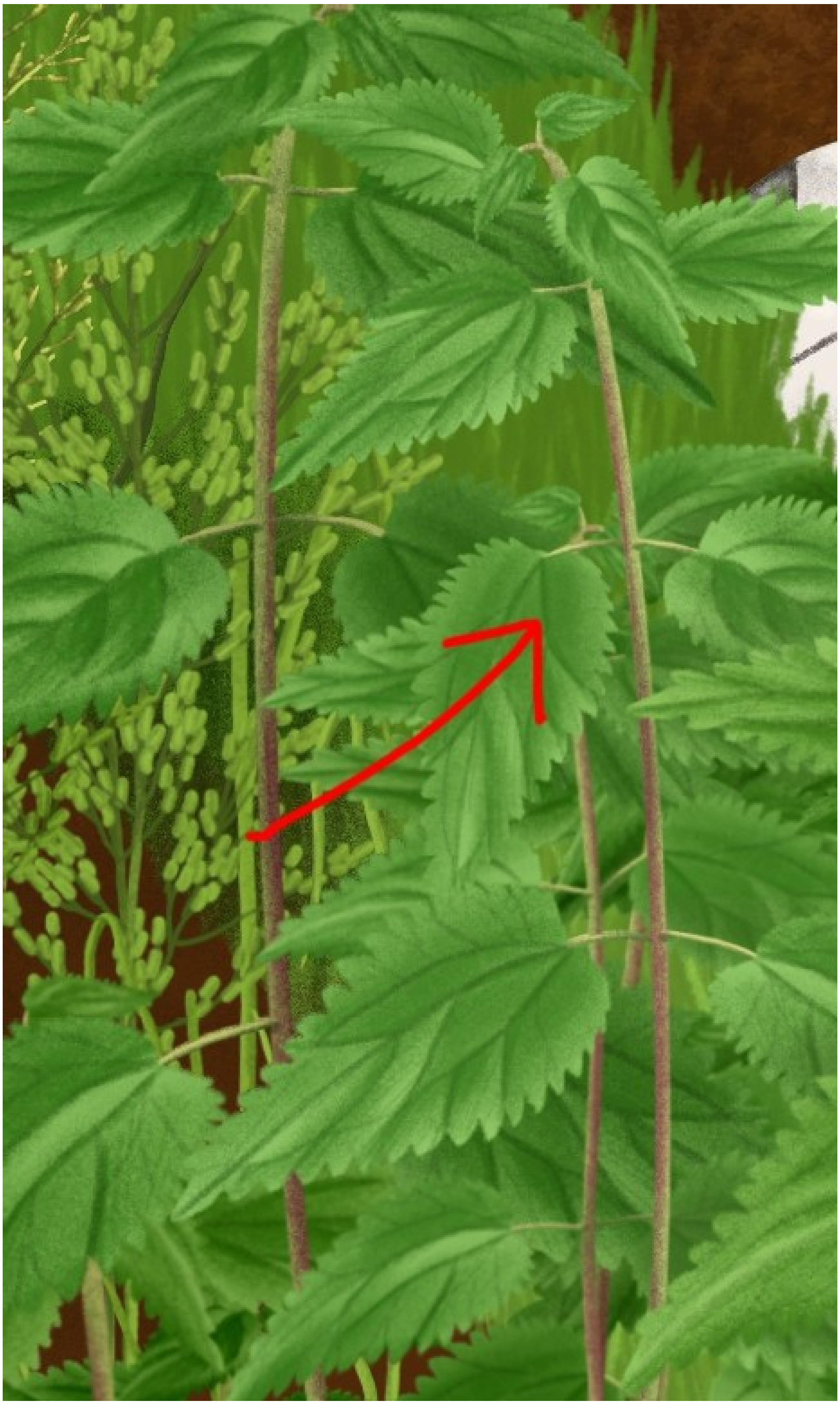

Please name the plant shown (if you know it).

Enter the name in the field.

18. 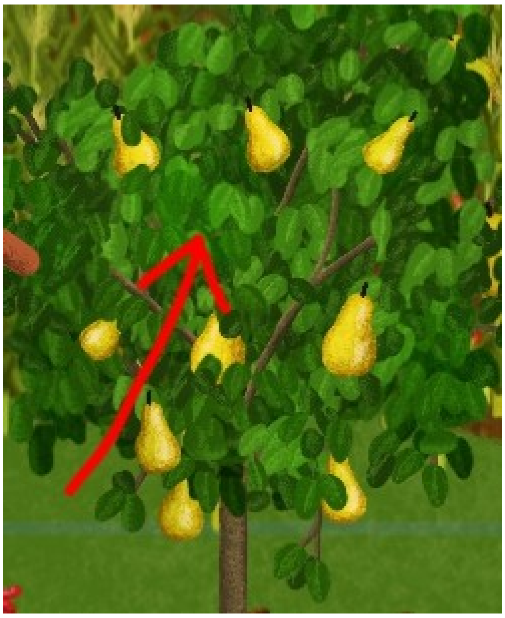

Please name the plant shown (if you know it).

Enter the name in the field.

19 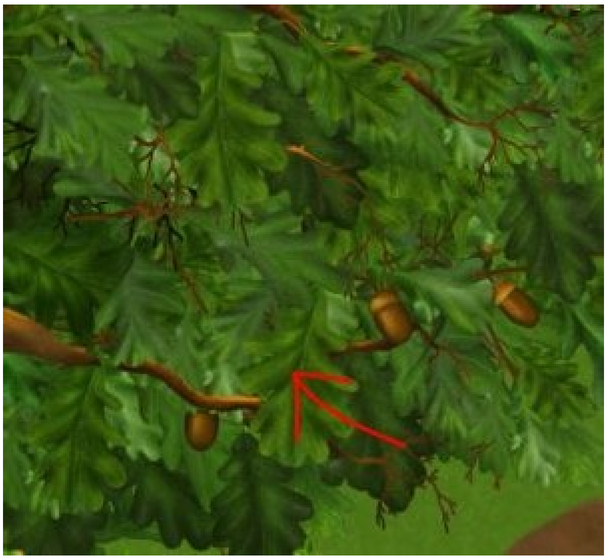

Please name the plant shown (if you know it).

Enter the name in the field.

20. 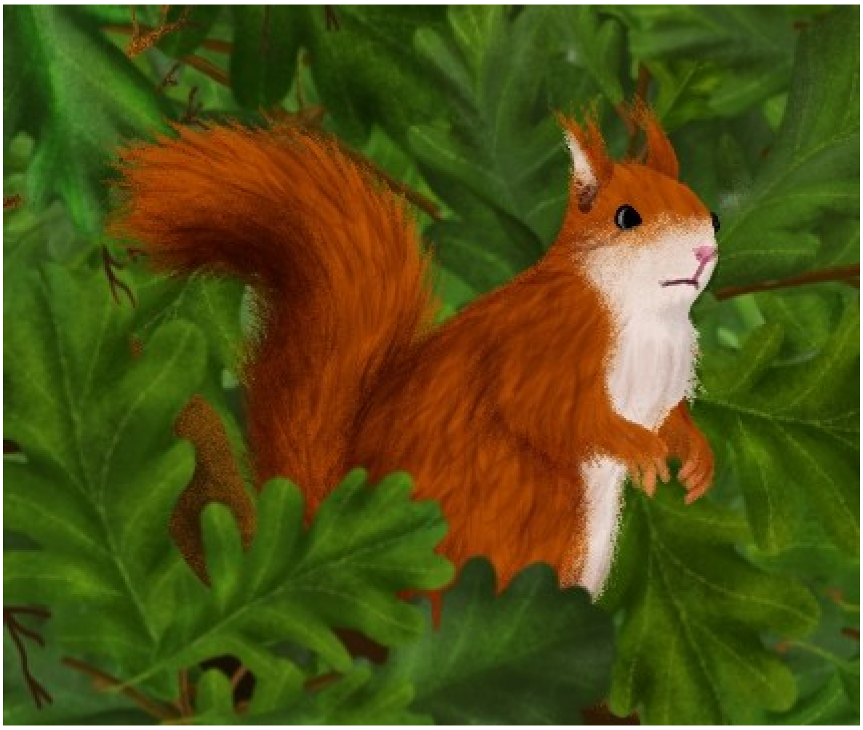

Please name the animal shown (if you know it).

Enter the name in the field.

21. 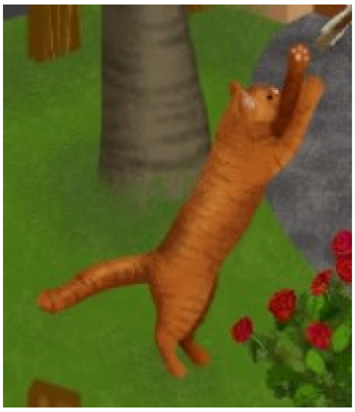

Please name the animal shown (if you know it).

Enter the name in the field.

22. 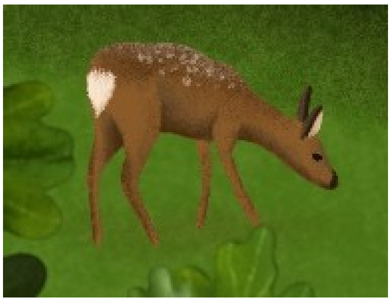

Please name the animal shown (if you know it).

Enter the name in the field.

23 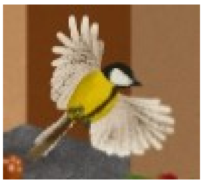

Please name the animal shown (if you know it).

Enter the name in the field.

24. 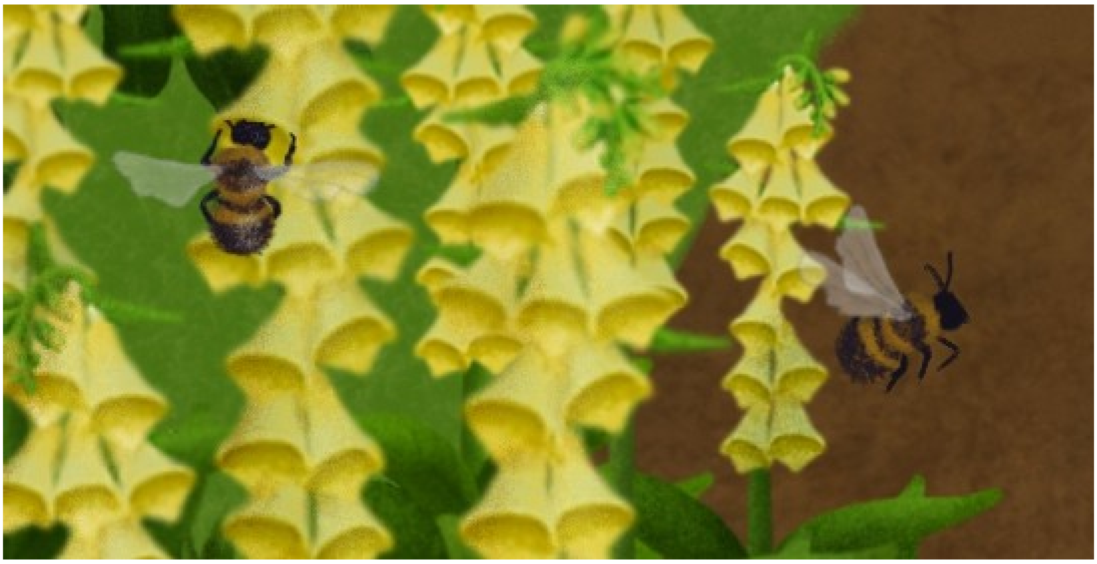

Please name the animal shown (if you know it).

Enter the name in the field.

25. 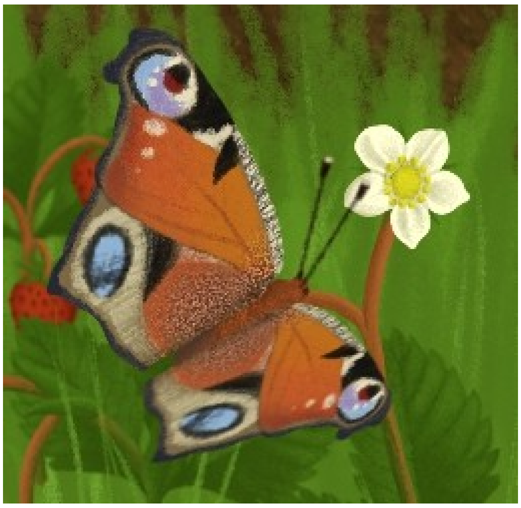

Please name the animal shown (if you know it).

Enter the name in the field.

26. 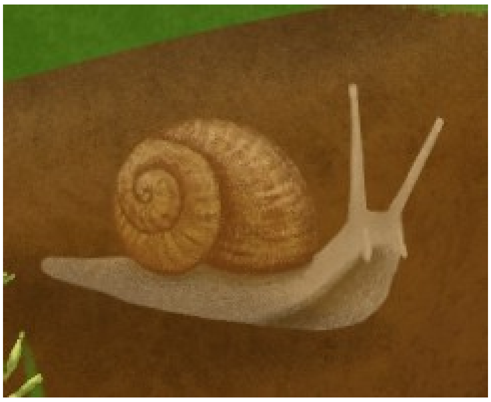

Please name the animal shown (if you know it).

Enter the name in the field.

27 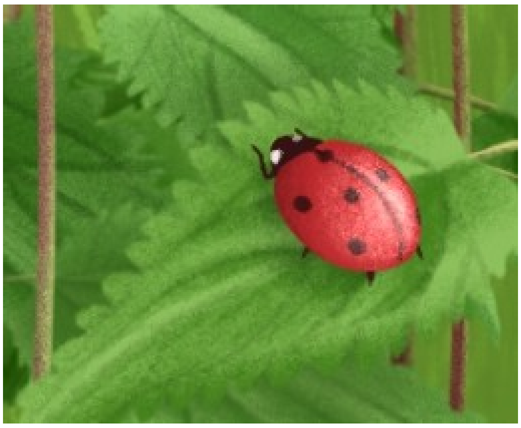

Please name the animal shown (if you know it).

Enter the name in the field.

28. 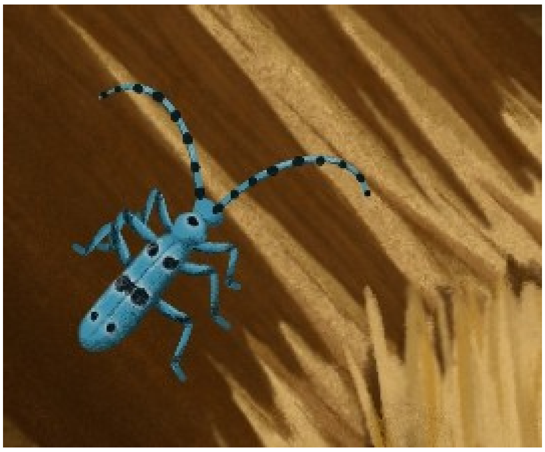

Please name the animal shown (if you know it).

Enter the name in the field.

29. 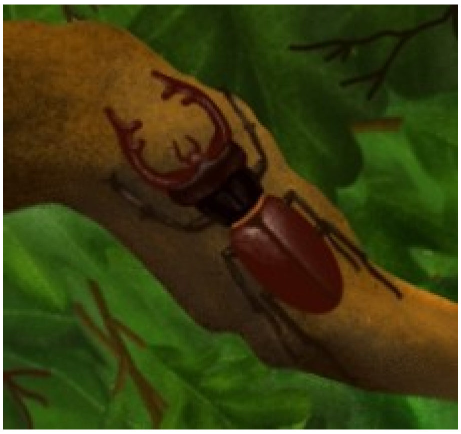

Please name the animal shown (if you know it).

Enter the name in the field.

Thank you for participating!

We would like to thank you very much for your help.

Your answers have been saved. You may now close your browser window.
