# Supplementary material for: Assessing attention towards plants: Development and first steps to the validation of the Hidden Object Picture Instrument (HOPI)
Source: PLoS One. 2026 May 21;21(5):e0349383. doi: 10.1371/journal.pone.0349383 (PMC13193508; doi:10.1371/journal.pone.0349383)
Supplement: S4 File — Labelled version of the image used in the HOPI. (PDF) [file pone.0349383.s006.pdf]

Explanation details (Part 1, left-hand side of the image):

**1**	**Spruce trees with spruce cones**
2	Wheat field with poppies
3	Combine harvester
4	Larger settlement (church, school, terraced houses, residential buildings, etc.)
5	Crow
6	Field
7	Maize field
8	Turn-off, access road to the house
9	Meadow (mainly dandelions, daisies, clover; also occasional yarrow, hogweed, buttercup, sorrel) + bees
10	Bed: sunflowers
11	Garden shed covered in ivy
12	Beehive, beekeeper in protective clothing (holding a honeycomb in a wooden frame)
13	Vegetable patch on the left: beetroot, peas, leaf lettuce Vegetable patch on the right: tomatoes, peppers, carrots
14	Stream, stones on the bank
15	Tree trunk (cut)
16	Small spruce saplings, including a spider's web and a spider (possibly a canopy spider)
17	Chanterelle
18	Tree fungus on a broken tree trunk
19	Alpine longhorn beetle
20	Peacock butterfly
21	Wild strawberries
22	Spruce cones
23	Fly
24	Wood fern
25	Ant trail
26	Moss and lichen
27	Toadstools
28	Dandelions and daisies
29	Forest path/forest road

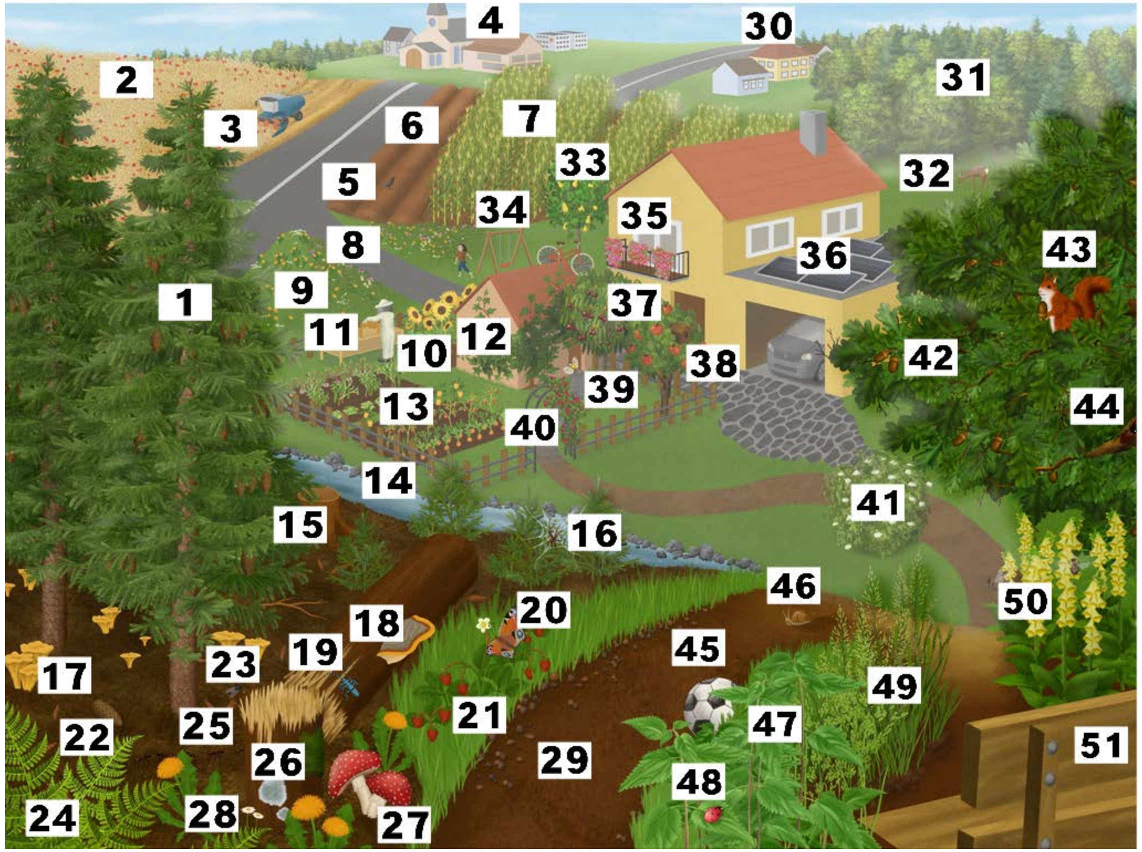

Explanation details (Part 2, right-hand side of the image):

30	Larger settlement (church, school, terraced houses, residential buildings, etc.)
31	Mixed forest: firs and beeches
32	deer feeding at the edge of the forest
33	Pear tree with several fruits
34	Bicycle, swing, child running
35	Balcony with geraniums
36	Solar panels
37	Cherry and apple trees, each bearing several fruits
38	Birdhouse or nesting box
39	Cat pouncing on a great tit
40	Rose arch
41	Elderberry bush
42	one or two distinctive branches of an oak tree, including acorns
43	Squirrel (partially hidden by leaves and/or branches)
44	Stag beetle
45	Forest path/forest road
46	Vine snail
47	Nettles, football pitch partially or largely obscured
48	Ladybird on a nettle leaf
49	Various grasses (wood sedge and/or wood bluegrass, golden oatgrass, smooth oatgrass)
50	Yellow foxglove with bumblebee(s)
51	Bench viewed from behind
