# Supplementary material for: Assessing attention towards plants: Development and first steps to the validation of the Hidden Object Picture Instrument (HOPI)
Source: PLoS One. 2026 May 21;21(5):e0349383. doi: 10.1371/journal.pone.0349383 (PMC13193508; doi:10.1371/journal.pone.0349383)
Supplement: S1 Table — (DOCX) [file pone.0349383.s008.docx]

Table S1: Land use in Austria – percentage of the Austrian national territory (rounded average value for the whole of Austria) (76,77).

| Type of landscape | percentage |
| --- | --- |
| Forest | 47.9% |
| Agricultural land (incl. arable land, permanent grassland and pastures) | 32.8 %. |
| Settlement area | 14% |
| Wasteland and water | 5.3% |
