# Supplementary material for: Assessing attention towards plants: Development and first steps to the validation of the Hidden Object Picture Instrument (HOPI)
Source: PLoS One. 2026 May 21;21(5):e0349383. doi: 10.1371/journal.pone.0349383 (PMC13193508; doi:10.1371/journal.pone.0349383)
Supplement: S2 Table — (DOCX) [file pone.0349383.s009.docx]

Table S2: Numbers of plant, animal, and fungi species in Austria (78–80).

| Plant species in Austria  (Ferns and flowering plants) | 3,462 |
| --- | --- |
| Herbs | 1,100 |
| Grasses | 350 |
| Shrubs | 250 |
| Trees | 125 |
| Ferns | 60 |
| Animal species in Austria | 54,125 |
| Insects | 40,000 |
| Birds | 213 |
| Mammals | 102 |
| Fish | 84 |
| Amphibians | 20 |
| Reptiles | 14 |
| Fungi species in Austria | 15,000 |
| Basidiomycetes | 5,000 |
