# Supplementary material for: Assessing attention towards plants: Development and first steps to the validation of the Hidden Object Picture Instrument (HOPI)
Source: PLoS One. 2026 May 21;21(5):e0349383. doi: 10.1371/journal.pone.0349383 (PMC13193508; doi:10.1371/journal.pone.0349383)
Supplement: Attachment — Submitted filename: PLOS_ONE_Response_letter_reviewers.pdf [file pone.0349383.s011.pdf]

Dear Reviewers,

Thank you for the positive feedback and helpful comments for correction and modification. The manuscript has been revised to address your comments: the changes are explained in detail in the table below and are marked in the manuscript with the help of the "track-changes" mode.

Best regards

The authors

Reviewer 1

#	Reviewer comments	Author response
1	The manuscript "Assessing Attention towards Plants: Development and Validation of the Hidden Object Picture Instrument (HOPI)" presents an innovative approach by developing a visual instrument to measure attention directed toward plants in complex scenes. This research addresses notable methodological gaps in the literature on plant awareness. However, the study demonstrates conceptual, methodological, and interpretative limitations that undermine its conclusions and require significant revision.	Thank you for this all-over positive feedback!
2	A primary concern involves the definition and operationalization of the construct "attention." While the manuscript distinguishes among attention, perception, and consciousness, its measurement relies primarily on free-listing metrics and eye-tracking parameters.<br><br>The free-listing approach primarily assesses memory and accessible vocabulary rather than immediate visual attention. Conflating delayed recall with attentional processing risks overstating what the instrument measures. Although the study reports correlations among salience, fixation time, and time to first fixation, it does not demonstrate that HOPI reliably isolates	Thanks to the reviewer for their insightful comments. Regarding the relationship between freely listing objects from memory and eye-tracking, the measures are meant to be complementary. This is now explained on p. 13: "Eye-tracking provided a straightforward measure of what participants paid attention to. In contrast, freely listed items represented what was remembered from the pictures instead of (only) what was looked at, in the first place. In fact, however, the two measures should be regarded as complementary and the freely listed items as, in fact, the probably slightly more relevant measure in the sense that the latter reflected a degree of awareness of the selected items that is not necessarily reflected in an eye movement. To note, firstly, the eye can be directed towards locations without selection of relevant information from these measures (as is sometimes observed in, e.g., change blindness, cf. Jensen et al., 2011). Secondly, participants can also execute covert selections by attention without moving the eye towards the relevant location (e.g., Posner, 1980). Thus, eye movements often but not always reflect what

#	Reviewer comments	Author response
	plant-specific attention or that salience directly indicates attentional priority.	is truly selected. Thirdly, and most important of the current study, though the protocol aims at an understanding of attention or information selection across the different levels of processing, from looking direction to ultimate encoding of selected objects, the later stages of encoded and successfully retrieved objects is the more relevant dependent variable in many applied contexts in which awareness or availability of the selected information from a substantial duration is critical (e.g., to allow appropriate conclusions about the lack or presence of particular plants and to act accordingly, e.g., to safeguard conditions for the presence of this plant). Therefore, both dependent variables - eye-movement measurements and freely listed objects - provide evidence about selected objects in a partly complementary fashion."<br><br>Jensen, M. S., Yao, R., Street, W. N., & Simons, D. J. (2011). Change blindness and inattentional blindness. Cognitive Science, 2(5), 529-546.<br><br>Posner, M. I. (1980). Orienting of attention. Quarterly Journal of Experimental Psychology, 32A(1), 3-25.<br><br>Regarding the failure to successfully validate the suitedness of the protocol for the study of plant selection in all respects, we kindly like to disagree: Just because one or the other of the dependent variables that we used fails to demonstrate an advantage or disadvantage of the selection of plants does not necessarily imply the insensitivity (or lack of validity) of the HOPI protocol. Instead, the corresponding data should be considered as potential exceptions to the phenomenon of plant unawareness. It is true that this same pattern of results might present in a different way with a different protocol, but withholding the present findings on the grounds of their (only partial) disagreement with some of the assumptions would add to a file-drawer problem. However, we agree with the upshot of these considerations and, thus, toned down our conclusions in the general discussion by pointing out that inconsistency of results across different dependent variables and protocols should be studied more systematically (e.g., the latter within the same participants) in future studies (see. p. 24).<br><br>Additionally, we added some paragraphs where we explain that our results show that students do have the vocabulary to name plants on a specific level but they do not, as long as they are not prompted to.

#	Reviewer comments	Author response
		"To explore how detailed students' species identification knowledge was and whether it correlates to where students looked at in the picture, as well as to which objects were recalled, after looking at the picture and recording eye-tracking data, students had to identify the 10 most salient animal and plant species (see Table 2) in the HOPI as accurately as possible. For this purpose, they were shown image sections of the hidden-object picture. The species identification was evaluated using a four-stage evaluation of the identification level (Table 1), from which the arithmetic mean value across all students was then calculated." (p 15)<br><br>Key findings reveal patterns connected to research questions 4, and 5, which focus on how students iden-tify species in the HOPI and how species identification relates to the results of the free-listing recall task. Despite students being able to identify plant species relatively well in the identification task (see Table 3), this did not translate into recall salience. In other words, the ability to name a plant species did not predict whether students recalled it during the free listing task. As the quite high identification scores show, students do not simply lack the language to name plants on a more specific level than "tree", but do not spontaneously attend to or recall them in the same way as animals or anthropogenic objects. Plants, instead, appeared in students' recall as "parts of the landscape" (e.g., "tree," "field") rather than as discrete species.<br>Additionally, this differentiation between general and specific terms used to describe plants (56) seems critical for interpreting the results and provides first answers to research question 6, addressing whether plants and animals are recalled at different levels of specificity. The salience of plants was relatively high compared to that of animals (Figure 4), but this result can be partially explained by very broad terms used to describe plants (e.g., tree, field, forest). Looking at plant salience at a more specific level reveals considerably lower levels of salience. However, the employment of general terms by both students and experts, primarily in the context of describing ecological regions and habitats, offers insight into the significance of plants in delineating specific biomes. This finding has the potential to inform future developments in the field of plant education by refining the focus from the analysis of broad habitats to the study of individual plant species. (p. 22-23)
3	The instrument's construction also merits critical evaluation. Although a controlled design allows precise	The reviewer makes a valid point, but we would like point to the fact that the content of a scene is not the only determinant of visual attention and visual

#	Reviewer comments	Author response
	manipulation of scene elements, it creates an artificial environment in which species distributions, proportions, and object quantities do not reflect those of realistic ecosystems.<br><br>The inclusion of non-coexisting species, disproportionate representation of taxonomic groups, and intentional enlargement of small objects result in a methodologically biased scenario.<br><br>Strong correlations between object size and salience indicate that the instrument primarily measures attention to size and visibility rather than to the plant category. Therefore, increased recall of larger plants should not be interpreted as evidence of heightened attention to flora as a cognitive category.	attention should be sensitive to mere visual characteristics, too. In this sense, the corresponding visual influences can be conceived of as sanity checks that demonstrate that our measures of visual attention were not artificially distorted in the current protocol. To the degree that the found content-driven differences between object categories (here, between plants and animals) were found despite the influences of mere visual factors demonstrates the robustness of the influence of the latter.<br>In setting without priming, size of plants does not seem to matter for attention with regard to attention blinks:<br><br>„In Experiment 1, adults sampled from a wide age range displayed inferior recall of plants in relation to animals even when the plant targets in the scene were taller, were prominent in the foreground and did not overlap with other items.<br><br>„In Experiment 2, we found that priming presented in the form of a botanically-relevant statement failed to attenuate the biasing effect of plant blindness on adults' recall performance. In Experiment 3, we ensured that the botanically priming stimulus was of the same modality and shared perceptual features with the target stimuli on our memory task (visual tree primes and visual tree targets) and we successfully uncovered evidence for the mitigation of plant blindness. The differences between the findings of Experiments 2 and 3 raise theoretical as well as methodological issues. The findings converge with broader evidence showing that priming effects are likely to occur at a pre-semantic rather than semantic level of processing, and fit with accounts arguing that humans have relatively distinct representational systems for verbal and visual modalities, with each of the systems having its own access routes and processing parameters „<br><br>Zani, G., & Low, J. (2022). Botanical priming helps overcome plant blindness on a memory task. *Journal of Environmental Psychology*, *81*. https://doi.org/10.1016/j.jenvp.2022.101808<br><br>Additionally, the HOPI does not include non-coexisting species, which is now added on page 12: The guiding principle when selecting the items was to choose well-known and clearly identifiable species, abundant in Austria (Central Europe), so that the picture only shows species that naturally inhabit the same habitat

#	Reviewer comments	Author response
		We removed the entire section regarding bivariate correlations and calculated a linear regression including also more eye-tracking parameters as independent variables (see p. 17).<br>To tackle RQ 3, we recorded the eye-tracking parameters *time to first fixation*, *total fixation duration*, *average fixation time*, and *number of fixations*. Subsequently, we used these eye-tracking parameters as well as the object size as independent variables to calculate a linear regression using the salience of an object as dependent variable (RQ 4). Additionally, the salience count (61) for each object (62) was calculated based on the free-listing recall task. A Spearman correlation was then computed between salience and identification test results (RQ 5).<br><br>This new analysis lead to the conclusion that object size does not correlate with salience. We adapted the results and conclusions, accordingly (see p. 19 and 22-23).
4	A further concern is the lack of equivalence between image versions used in different studies. The removal of the cat, which the manuscript identifies as necessary to reduce emotional priming, substantially alters the visual composition and undermines comparability across studies. For an instrument intended to achieve psychometric validity, modifying key items between applications is problematic, as it impedes the establishment of reliability and stimulus invariance. Additionally, the inclusion of a highly salient domestic animal in Study 1 may have introduced biases, complicating direct comparison with Study 3.	We agree with the reviewer and added the corresponding information to the limitations (p. 24): " While we advocate the view of the HOPI as one singular protocol, we want to point out that slightly different versions of the HOPI were used in the sub-studies. These changes were part of the HOPI iteration process to refine it. To address this aspect, we aimed to be as transparent as possible in highlighting how we have applied the HOPI and which adjustments have been made. Small changes, e.g., adjusting sizes, removing a prominent object like the cat, or how long participants look at the picture, can influence the outcomes of the HOPI. Therefore, the different versions of the HOPI protocol in this contribution require a selection on the part of the future user. Furthermore, all analyses presented in this paper for different versions of the HOPI should be applied to the final one.<br>The current explanations of the different results achieved with the protocol's versions, as well as the motivations for using alternative versions of HOPI in the present study hopefully provide some direction for the user about which version to employ for a particular purpose. In addition, in future studies, different versions of the protocol should be compared within the same participants, so as to clarify which of the found differences were due to the alternative protocols and which were due to the different samples. Moreover, the test-retest-reliability of the HOPI has to be calculated in future research."
5	The interpretation of differences between experts and students also warrants scrutiny. While analyses indicate that experts employ specific terminology and students	This is a wonderful comment, and we incorporated in the following manner in the current manuscript: “Both the free-listing recall task and the eye-tracking provided important information regarding human observers’ selection of plant-specific

#	Reviewer comments	Author response
	use more generic descriptions, this likely reflects disparities in vocabulary and taxonomic knowledge rather than differences in directed attention. Although the manuscript acknowledges this limitation, it nonetheless concludes that HOPI differentiates levels of attention, a claim that is not sufficiently supported given the lack of control for linguistic variables. Reliance on free listing as a primary measure increases the risk of conflating knowledge and language proficiency with perceptual processes. Furthermore, the 30-second exposure time limits the range of attentional trajectories and encourages rapid, focused scanning of large, contrasting, or emotionally salient objects. The species identification test, which uses fragments from the original image, does not distinguish between superficial visual recognition and prior knowledge. While students may identify species when prompted, this does not necessarily indicate spontaneous attention during image observation.	information. They should be seen as complementary methods of gaining insights into different facets of attention. Each method has its particular advantages and draw-backs, as the study shows. For example, we already mentioned that looking behaviour alone, which can be measured by eye-tracking, does not necessarily provide much insight into the degree of memory consolidation (48) and/or the awareness that follows from looking at an object. This was confirmed in our study through the lack of any significant correlations between eye-tracking parameters and object salience. Our study also indicates some shortcomings of the free-listing recall task. For example, group differences between experts and students in the free-listing recall task partially reflected differences in background knowledge and command of the relevant jargon. Based on the advantages and drawbacks of each method, we advocate using these methods in a complementary way and avoiding focusing solely on either eye movements or recall abilities for a full understanding of attention to plants and plant aware-ness." This is now explained on p. 24.
6	Analytically, the study relies primarily on bivariate correlations and does not employ multivariate models that could disentangle the effects of size, familiarity, position, contrast, color, and taxonomic category. In the absence of such controls, the resulting inferences are vulnerable to confounding variables. Additionally, the study does not provide evidence of the instrument's test-retest reliability or compare it with other established measures of plant attention, thereby weakening the assertion that HOPI is a validated instrument.	We thank the reviewer for this valuable comment that was very helpful to adapt our analytical strategy. Following the recommendations, we removed the entire section regarding bivariate correlations and calculated a linear regression including also more eye-tracking parameters (see p. 17).<br>"To tackle RQ 3, we recorded the eye-tracking parameters *time to first fixation*, *total fixation duration*, *average fixation time*, and *number of fixations*. Subsequently, we used these eye-tracking parameters as well as the object size as independent variables to calculate a linear regression using the salience of an object as dependent variable (RQ 4). Additionally, the salience count (61) for each object (62) was calculated based on the free-listing recall task. A Spearman correlation was then computed between salience and identification test results (RQ 5). "<br><br>We also adapted the resulting conclusions, accordingly (see p.22-23).<br><br>Furthermore, the reviewer is right in that we did not use a classically controlled laboratory design in which each object was presented equally likely at each location, in each color, contrast, etc. To note, however, some of these aspects of control are

#	Reviewer comments	Author response
		also not necessarily desirable for an ecologically plausible stimulus. For instance, arbitrary coloring of objects would create degrees of color-object inconsistencies in the pictures compared to the experience outside and before the experiment (cf. Price & Humphreys, 1989). This is known to affect attention (e.g., Frey et al., 2008) and memory (e.g., Wichmann et al., 2002) in its own right and, most importantly, would corrupt the link between the protocol and real-world applications. The same applies to the choice of object locations that are often linked to objects in real-world environments (e.g., Wolfe, 2021). In addition, we agree with the reviewer that some of these confounding differences might be important and others not. Thus, the study of the corresponding differences is interesting, but for the above argument, it has not been a priority in the present study. Again, we added the corresponding limitation in our discussion-section.<br><br>“The current explanations of the different results achieved with the protocol's versions, as well as the motivations for using alternative versions of HOPI in the present study hopefully provide some direction for the user about which version to employ for a particular purpose. In addition, in future studies, different versions of the protocol should be compared within the same participants, so as to clarify which of the found differences were due to the alternative protocols and which were due to the different samples. Moreover, the test-retest-reliability of the HOPI has to be calculated in future research.”<br><br>Finally, we agree with the reviewer that even the current study is a compromise between real-world ecology and control in the sense that we used a painting of the stimuli that only partially matches the visual conditions that hold in the real-world. In this respect, we kindly wanted to remind the reviewer, however, that it is probably not easy to come up with an entirely realistic real-world image of the critical objects because of the natural variability of all objects involved in terms of visual perspectives, seasons, lighting conditions (e.g., time of day), or exact object/scene compositions. In other words, each image selection, even that of a photorealistic depiction of the objects, comes with a degree of uncertainty regarding its ecological validity. At this point, we would argue for the start of the current research line, despite not having solved each of these associated problems in advance, just as it has been done successfully in other areas of research in which visual confounds and the lack of control thereof might have compromised the soundness of some of the

#	Reviewer comments	Author response
		conclusions (e.g., for the International Affective Picture System, Delplanque et al., 2007).<br><br>Delplanque, S., N'diaye, K., Scherer, K., & Grandjean, D. (2007). Spatial frequencies or emotional effects?: A systematic measure of spatial frequencies for IAPS pictures by a discrete wavelet analysis. Journal of Neuroscience Methods, 165(1), 144-150.<br><br>Frey, H. P., Honey, C., & König, P. (2008). What's color got to do with it? The influence of color on visual attention in different categories. Journal of Vision, 8(14), Article 6.<br><br>Price, C. J., & Humphreys, G. W. (1989). The effects of surface detail on object categorization and naming. The Quarterly Journal of Experimental Psychology, 41(4), 797-828.<br><br>Wichmann, F. A., Sharpe, L. T., & Gegenfurtner, K. R. (2002). The contributions of color to recognition memory for natural scenes. Journal of Experimental Psychology: Learning, Memory, and Cognition, 28(3), 509-520.<br><br>Wolfe, J. M. (2021). Guided Search 6.0: An updated model of visual search. Psychonomic Bulletin & Review, 28(4), 1060-1092.
7	The conclusions presented in the manuscript appear to extend beyond the data. The assertion that the instrument measures "spontaneous attention" is premature, as most observed differences are attributable to basic visual factors or participants' linguistic repertoires. The educational claim that HOPI could function as a diagnostic or evaluative tool for pedagogical interventions is also unsupported, since the study did not assess intervention effects or demonstrate the instrument's sensitivity to temporal or contextual changes in attention patterns.	In contrast to the reviewer's view, we hope that the current study provides first insights into the heuristic value of our protocol. However, the reviewer is right in terms of the lack of some of the mentioned characteristics, and the corresponding conclusions have been toned down and we removed the term "spontaneous attention". See also p. 26 for an example:<br><br>"One of the potential implications of this study lies in education. Since attention is a prerequisite for learning, measuring how students attend to plants provides a critical diagnostic for plant awareness education (23,74). The HOPI can serve as an instrument for evaluating the impact of pedagogical interventions designed to foster plant awareness. For example, structured activities that prime learners to attend to specific plant traits could shift recall patterns from general to specific categories, gradually building more precise and meaningful plant knowledge. Our findings also show that attention can be promoted: experts' higher salience for plants and their

#	Reviewer comments	Author response
		more specific naming demonstrate that experience and training reshape attentional priorities.”
8	The manuscript requires conceptual clarification, as it alternates between presenting the instrument as a measure of "spontaneous attention" and as an indicator of the ability to recognize or identify plants as a biological category. Distinguishing between attention or perception and conceptual knowledge (or "botanical literacy") is central to current debates on "botanical blindness." The following article is recommended to support the authors’ theoretical reflection: https://www.scielo.br/j/abb/a/VcPZ6cyqgXVBWKmYbq68Hqx/?lang=en	Thanks to the reviewer for their comment and the hint to this really interesting theoretical paper. We have clarified this point (see also our response to comment 1 above) and included the recommended reference on page 5
9	This study has the potential to make a significant contribution to the field, but it requires substantial revisions to address these weaknesses. A more rigorous definition of what HOPI measures and a clearer delineation of valid inferences from its application are necessary.	Thank you again for your valuable comments We have toned down our conclusions and clarified the limitations of the study.

Reviewer 2

#	Reviewer comments	Author response
1	The manuscript presents a relevant and timely contribution by addressing the issue of attention toward plants, a topic of growing interest within environmental education, cognitive science, and perception studies. By proposing an instrument to investigate visual attention to plants, the study engages with an important and underexplored dimension of human–environment interactions. Overall, the work demonstrates methodological care and an ambitious scope; however, several conceptual and methodological aspects would benefit from further clarification to strengthen the coherence and impact of the study.	Thank you for this all-over positive feedback!
2	1. Introduction and Theoretical Framing<br><br>The introduction provides a solid overview of the relevance of plant awareness and its implications for education and environmental perception. Nevertheless, the transition from the general discussion of "plant blindness" to the specific proposal of a new measurement instrument could be more clearly articulated. While the manuscript argues for the importance of assessing attention to plants, it does not fully clarify why existing approaches are insufficient, nor how the proposed instrument fills this gap in a distinct and necessary way.<br>In addition, although the concept of priming is mentioned, the rationale for deliberately avoiding priming in the study design could be more explicitly developed. A clearer explanation of why the absence of priming is methodologically important for assessing spontaneous attention would strengthen the theoretical grounding of the study.	Thank you for these comments, we adapted our introduction on p. 6:<br><br>"The existing literature highlights a critical gap: although attention towards plants is recognised as a key component of plant awareness, there is no widely validated, scientifically grounded instrument to measure it directly. Some existing instruments focus only on the difference between mammals and plants (56,58). Other studies on plant awareness often rely on self-reports or general recall tests without stimuli, (e.g. 59), which may not accurately reflect real-time attention processes. Furthermore, previous re-search has largely addressed attention towards plants implicitly, without systematically operationalising it as a measurable construct. Developing an instrument to measure attention towards plants is important for at least two reasons. Firstly, from a research perspective, it would allow a more precise examination of the cognitive and perceptual processes underlying plant awareness, thereby providing a stronger theoretical foundation for interventions. Secondly, from an educational perspective, such an instrument could serve as both a diagnostic and evaluative instrument, helping educators identify baseline attention levels and assess the impact of plant-focused teaching strategies or interventions."<br><br>Regarding priming, we adapted our text on p. 8:<br>"Since the most important question for this study was to find out which plant-related information was recalled and how (RQ 1), we decided to use an open recall task as established method (29), and to collect data via free listing. Free listing is an

#	Reviewer comments	Author response
		effective method in a recall task because it avoids the use of predefined terms. Instead of constraining responses to a checklist, it invites participants to freely list all items they remember, yielding richer, more authentic insights into their mental representations (60). This open-endedness captures both shared and unique elements of recall, and it mirrors the way information is naturally stored and retrieved in memory. This approach also reduces researcher bias, as data are de-rived directly from participants' language rather than being filtered through predetermined categories or (61). Therefore, we decided not to give a plant-specific prompt or to use semantic or perceptual priming (e.g. 54). This aspect is particularly important for assessing whether students remember the objects on a different level of specificity (RQ 6)."
3	2. Objectives and Conceptual Coherence<br><br>The manuscript states that its objective is to develop and validate an instrument to assess attention to plants. However, throughout the text, the emphasis appears to be placed primarily on the development and application of the instrument, whereas the process of validation is less clearly articulated. As a result, there is some tension between the stated aims and the outcomes presented.<br><br>Clarifying whether the study's primary goal is the validation of a new instrument or the exploratory demonstration of its potential would improve the internal coherence of the manuscript. Explicitly outlining the criteria used to assess validity would further strengthen this section.	To follow these line of argumentation, we decided to separate our goals into seven distinct research questions, that are outlined on p. 7-8:<br><br>"The study presented has two main objectives: (1) to develop a new, validated instrument for measuring attention towards plants. (2) To establish a transparent and replicable methodology for designing ver-sions of such Hidden Object Picture Instruments. , This methodology will facilitate future research and educational application studies investigating attention towards plants and other organisms<br>To address these objectives, this work explores the following research questions:<br>(RQ 1) Are certain objects in the HOPI (plants, animals, non-living and anthropogenic objects) recalled differently when participants have to report what they saw in the picture?<br>(RQ 2) Are the species depicted in the HOPI recognisable, and do participants recognise them?<br>(RQ 3) Do specific object classes in the HOPI (plants, animals, non-living and anthropogenic objects) attract attention in different ways when participants look at the picture?<br>(RQ 4) Do specific properties of the depicted objects in the HOPI (e.g., size) correlate to how well they are recalled?<br>(RQ 5) Do participants recall species previously known to them better?<br>(RQ 6) Are specific object classes (plants, animals, inanimate and anthropogenic objects) recalled on a different level of specificity?<br>(RQ 7) Does the HOPI allow to discriminate between participant groups differing in their recall of plants?

#	Reviewer comments	Author response
		While research question 1 relates to the potential of the HOPI for exploring a person's attention towards plants, research questions 2–7 contribute to the validation of the HOPI and to ensure that it is an appropriate tool to explore research question 1.<br><br>In the subsequent sections of the text, we always refer to these research questions to make clearer, which part of our manuscript follows which purpose, e.g. p. 21-22:<br><br>"Regarding research question 1, which investigates differences in object recall, the HOPI was effective in identifying salient objects (such anthropogenic/non-living objects for students or even plants for botany experts). The depicted plant and animal species were readily identifiable without problems (research question 2), as confirmed by expert validation. This finding eliminates the possibility that the indiscriminateness of the depicted plants accounts for the low plant recall observed among students. Addressing research question 7, the instrument demonstrated known-groups validity: students and botanical experts differed significantly in their attentional allocation in the free-listing task, with experts showing the high-est salience for plants, while students showed the highest salience for human/non-living objects. Such distinctions align with theoretical expectations and lend support to the HOPI's ability to assess what is intended (71). The differences between the groups also underscore the role of top-down, culturally mediated knowledge on attentional bias (14) in contrast to mere bottom-up attentional processes, in which salient visual features solely direct gaze (36)."
4	3. Methodological Design and Construction of the Instrument<br><br>The methodological section provides a detailed description of the procedures used to construct and apply the visual stimulus. However, certain aspects of the design would benefit from greater transparency. In particular, the rationale underlying the selection, proportion, and spatial arrangement of elements within the visual stimulus is not always clearly articulated.<br><br>Although the manuscript acknowledges that adjustments were made to the image throughout the	Thank you for this valuable comment.<br><br>We refined our reasoning for the picture composition on p. 10-12, e.g. "By adding these items to the HOPI, the sketch showed a slight deviation from the previously researched numerical ratios of the organism groups to be represented. However, a deliberate decision was made in favour of these optimisations, as the HOPI still corresponds to a representation of a Mid-European land-scape. It should also be mentioned that it was not possible to display all items in their correct proportions, as otherwise some of them would no longer have been recognisable due to their tiny original size. In this case, we decided to give more weight to the recognisability of the objects than to naturalistic depiction, leading to positioning the small species rather in the foreground and larger species rather in the background. Furthermore,

#	Reviewer comments	Author response
	development process, these modifications are not consistently linked to explicit methodological or theoretical criteria. As a result, it remains somewhat unclear which aspects of the final stimulus are essential to the method and which are contingent on the specific context of this study.<br><br>Furthermore, while the inclusion of multiple participant groups is a strength, the criteria guiding sample size selection and group composition are not fully explained. Clarifying whether these choices were driven by statistical considerations, practical constraints, or theoretical assumptions would enhance the transparency and reproducibility of the study.	we decided to take into account flight distances of animals, resulting in putting insects in the foreground an the mammals and birds rather in the background.<br><br>We also tried to make clearer, why we excluded or changed some objects (p. 17):<br><br>"To evaluate the known-groups validity (71) of our assessment instrument, we compared scores from students attending two distinct school types, as well as a group of botanical experts. This approach test-ed the instrument's ability to discriminate between groups theoretically expected to differ in their knowledge of and, thus, attention towards plants. For study 3, we used a slightly modified version of the picture, which is the final version of the Hidden Object Picture Instrument (Figure 3). The following minor adjustments were made:<br>• we removed the cat in the centre of the picture to prevent excessive visual attention through a companion animal in movement (cat jumping for the Great tit)<br>• we reduced the size of the snail in order to get more realistic proportions<br>• we reduced the colour saturation of parts of the picture that were in the background (i.e., farther away) to evoke a more realistic perception of the landscape, because colour saturation helps us to perceive distance and thereby relative object size."<br><br>Our sample sizes in study 1 and 3 were determined primarily by practical reasons rather than statistical or theoretical ones. We added explanations to the text (p. 18)<br><br>To encompass the broadest possible spectrum of students as possible, participants included 427 students from two urban middle schools (grades 5–8; 47% male, 53% female, 0% diverse; Mage = 13.25 ± 2.11), and 388 students from three rural Naturparkschulen (grades 5–8; 47% male, 43% female, 0% diverse; Mage = 12.25 ± 1.26). The number of students were primarily determined by the teachers who consented to administer the tool during their lessons. In order to broaden the comparison, we again included a group of experts from the Austrian Flora Research Association (http://www.flora-austria.at). A total of 66 members (Mage = 53.1 ± 19.3, 55% male, 45% female, 0% diverse) voluntarily participated following a newsletter invitation via email. All participants again received the free-listing recall task: "What did you see in the picture? Write down a list of all objects in the picture you can remember." Subsequently, we calculated the salience for each object as well

#	Reviewer comments	Author response
		as the mean personal salience for the categories plants, animals, and human/non-living objects.<br><br>In study 2, we followed the recommendations of<br>Hoogerbrugge, A. J., Hooge, I. T. C., Hessels, R. S., & Strauch, C. (2025). When is enough enough? Empirical guidelines to determine participant sample size for scene viewing studies. *Behavior Research Methods*, *57*(9), 241.<br>https://doi.org/10.3758/s13428-025-02754-8<br><br>We added this reference also on p. 17.
5	4. Results and Their Relationship to the Study Objectives<br><br>The results section presents informative patterns regarding visual attention, including differences between participant groups and between categories of visual elements. However, these findings appear to address descriptive questions about attention more directly than they substantiate the validation of the proposed instrument.<br><br>In other words, while the results demonstrate that the instrument can generate meaningful data, they do not fully establish how or why these outcomes confirm the instrument's validity. A clearer articulation of how the observed results support the proposed measurement framework would strengthen the link between empirical findings and the study's stated objectives.	See also answer to comment n°3 above. To follow these line of argumentation, we decided to separate our goals into seven distinct research questions, and, subsequently,<br>refer to them to make clearer, which part of our manuscript follows which purpose, e.g. p. 14:<br><br>"In this study, we examined how students visually explored the picture. For this purpose, we tested whether students looked earlier or longer at plants or animals (RQ 3) in the hidden object picture. Additionally, we tested whether students looked first at large items or species they already knew (RQ 4), and whether species they already knew were better recalled (RQ 5)."<br><br>or p. 19<br><br>"Study 1 resulted in a piloted high-resolution hidden object picture presenting an idealistic central European landscape (Figure 2). The students' (n = 218) results of the free-listing recall task were used to calculate the salience of each object in the HOPI (RQ1; see Table 2 for the top-10 species). All tested species were clearly identifiable in the expert validation (RQ 2)."<br><br>"For three of these parameters (time to first fixation, total fixation duration, and number of fixations), ANOVA showed no significant differences between their mean values for plants, animals, and anthropogenic/non-living objects. The only eye-tracking parameter showing differences in the ANOVA was average fixation time (RQ3); students looked significantly ($p < .05$) longer at the animals than at plants and anthropogenic/non-living objects. Linear regression showed no significant results for

#	Reviewer comments	Author response
		the four eye-tracking parameters as well as the size of an object as predictors for the salience of an object (RQ4)."
6	5. Discussion and Implications<br><br>The discussion highlights the potential relevance of the instrument for educational and research contexts, but it would benefit from a more explicit reflection on the methodological implications of the findings. In particular, the manuscript would be strengthened by a clearer discussion of:<br><br>- which components of the instrument are essential for its effective application;<br>- which elements may be adapted or modified in future studies;<br>- how researchers might replicate or extend the method in different cultural or ecological contexts.<br><br>Addressing these points would help clarify the scope and limitations of the proposed approach and enhance its usefulness for future research.	We overworked the discussion-section thoroughly and added comments regarding the central components as well as future modifications<br><br>p. 23<br>"A second major contribution of this study is the provision of a template for creating additional HOPIs to study attention towards other taxonomic groups, as well as within different cultures and regions. The instrument was developed and refined in a multimethodological multi-study approach. Species selection was based on Austrian biodiversity data, while design choices (e.g., inclusion of flowering plants, balanced representation of taxa, size adjustments for recognisability) ensured both realism and identifiability. Each adaptation – from hand-drawn sketches to the final digital picture – was systematically documented, allowing replication and adaptation for other contexts. In total, more than 1,000 students and more than 60 botanical experts were involved in this iterative process, combining ecological realism with methodological rigour. This approach strengthens the credibility of the present instrument and, after some more validation steps, similar procedures could be used, for instance, to develop HOPIs focus-ing on insects or fungi, thereby broadening the scope of research on attention to (aspects of) biodiversity."<br><br>p. 24<br>"For developing such effective Hidden Object Picture Instruments, several components are essential: (1) a carefully designed drawing (rather than a photograph) that allows tight control over stimulus content and object placement; (2) a conceptual ecological frame that defines the landscape type (e.g., an "average" regional habitat) and guides the inclusion of organism groups and approximate relative frequencies; (3) selection of well-known, clearly identifiable items that plausibly co-occur in the depicted habitat, with recognisability prioritised over strict naturalistic proportions when necessary; and (4) a high-resolution professional realisation based on a detailed sketch and explicit species-specific instructions (names, reference images, and diagnostic features) to ensure unambiguous identification.

#	Reviewer comments	Author response
		At the same time, multiple elements can be adapted in future studies: researchers may vary the season (to adjust stimulus variety and colour constraints), species lists and group ratios (balancing realism with feasibility), foreground–background placement rules (e.g., size scaling, flight-distance logic, salience control), and visual tuning such as colour saturation and removal of attention-grabbing distractors. To replicate or extend the method across cultural or ecological contexts, researchers can keep the *workflow* constant—define a locally meaningful "typical" habitat using regional land-use and biodiversity information, choose culturally familiar and locally abundant species that co-occur, and document all design decisions – while substituting region-specific content (e.g., different taxa, built-environment features, or landscape structures). This approach enables comparable instruments across settings, and also allows systematic cross-cultural tests by holding design principles stable while varying only the ecological and cultural parameters of the scene."<br><br>p. 25<br>"The current explanations of the different results achieved with the protocol's versions, as well as the motivations for using alternative versions of HOPI in the present study hopefully[SE60.1] provide some direction for the user about which version to employ for a particular purpose. In addition, in future stud-ies, different versions of the protocol should be compared within the same participants, so as to clarify which of the found differences were due to the alternative protocols and which were due to the different samples. Moreover, the test-retest-reliability of the HOPI has to be calculated in future research."<br><br>"Furthermore, using common human companion animals as objects in such research tools can be a limita-tion, as they might draw more attention from the viewer because[SE66.1] they have an affective impact on their attentional range. In study 1, we found slightly higher salience for animals than in comparison to[SE67.1] study 3 (see Table 2 and Figure 3). This difference may be attributed to the removal of the cat, which was sitting in the centre of the picture in the first two studies, and was omitted in study 3. This central position and potential priming role of the cat was the main reason for excluding it for the final HOPI in study 3. This aspect of priming (73) by removing or adding elements in the picture must be subject for further studies."<br><br>p. 27

#	Reviewer comments	Author response
		"Ultimately, this study represents a first step towards systematically assessing attention towards biodi-versity. The HOPI not only highlights disparities in attention towards different groups of organisms but also provides a scalable model for future biodiversity awareness research. Developing comparable in-struments for insects, fungi, or aquatic organisms would allow researchers to map attentional hierarchies across taxa and to design interventions that address imbalances. It is important to note that advancing plant awareness should not occur at the expense of other organism groups[SE80.1]. Rather, fostering attentiveness to plants should be seen as a gateway to broader biodiversity awareness, enabling learners to appreciate organisms as interconnected components of ecosystems rather than isolated entities."
7	6. Concluding Remarks<br><br>In summary, the manuscript presents a promising and innovative approach to studying attention to plants. However, to fully realize its potential contribution, greater clarity is needed regarding the relationship between the study's objectives, methodological choices, and conclusions. Strengthening the discussion around validation, methodological transparency, and transferability would significantly enhance the scientific robustness and broader applicability of the work.	Thank you again for your helpful comments.
