# Supplementary material for: Assessing attention towards plants: Development and first steps to the validation of the Hidden Object Picture Instrument (HOPI)
Source: PLoS One. 2026 May 21;21(5):e0349383. doi: 10.1371/journal.pone.0349383 (PMC13193508; doi:10.1371/journal.pone.0349383)
Supplement: Attachment — Submitted filename: Answer Letter Reviewers_Revision 2.pdf [file pone.0349383.s012.pdf]

Dear Reviewers,

Thank you again for the positive feedback and helpful comments for correction and modification. The manuscript has been revised to address your comments: the changes are explained in detail in the table below and are marked in the manuscript with the help of the "track-changes" mode. We changed the order of the comments with regard to the effort necessary to address them, starting with those slight changes that concerned only one or two sentences, going on to those requiring some more extensive restructuring.

Best regards

The authors

#	Reviewer comments	Author response
1	Thank you for the opportunity to review the new version of the manuscript. I appreciate the authors' careful consideration of the comments raised in the previous round of review and their efforts to address those points in the current version. The manuscript has clearly benefited from these revisions and has improved in several important respects. At the same time, this new version has also raised a few additional points that, in my view, would further strengthen the clarity and conceptual coherence of the study. I outline these suggestions below for the authors' consideration.	Thank you for this positive feedback!
2	The citation format in the following sentence is somewhat unclear: "Charismatic megafauna, particularly large mammals, often dominate conservation priorities and public awareness (12,13). (14,15). Recent estimates suggest that nearly 40% of vascular plant species – and three-quarters of undescribed species – are threatened with extinction (16)." In particular, the segment "(12,13). (14,15)." appears inconsistent. I would kindly suggest revising this part to ensure clarity and alignment with the journal's referencing style.	Corrected to: "Charismatic megafauna, particularly large mammals, often dominate conservation priorities and public awareness (12,13,14,15)."
3	In the sentence "This methodology will facilitate future research and educational application studies investigating attention towards plants and other organisms", please include a period at the end of the sentence.	Done.

4	In the Methods section, the sentence "This approach also reduces researcher bias, as data are derived directly from participants' language rather than being filtered through predetermined categories or (61)." appears to be incomplete. I would kindly suggest revising this part for clarity.	Changed to: "This approach also reduces researcher bias, as data are derived directly from participants' language rather than being filtered through predetermined categories (61)."
5	I would kindly suggest revising the following sentence for clarity: "Subsequently, three main categories were formed for comparing the results of plants with other objects: plants, animals, and human/non-living objects, with the last category encompassing everything that does not fall into the other three." As currently phrased, it is somewhat unclear, since three categories are defined, but the last category appears to include everything that does not fall into the other two.	Changed to: "Subsequently, three main categories were formed for comparing the results of plants with other objects: plants, animals, and human/non-living objects. The last category includes both living (child, beekeeper) and inanimate natural (stream, stone), as well as human-made (house, football, bench) objects."
6	I would like to highlight a potential inconsistency between the formulation of RQ4 in the introduction and its operationalisation in the Methods section. In the introduction, RQ4 is defined as examining whether specific properties of the depicted objects (e.g., size) correlate with how well they are recalled. However, in the Methods section, RQ4 is described as testing whether students "looked first at large items," which appears to relate to visual attention (eye-tracking measures) rather than recall. This difference makes it somewhat unclear how RQ4 is being addressed. I would kindly suggest clarifying whether RQ4 is intended to focus on recall, visual attention, or a relationship between the two, and ensuring consistency between the research question and its methodological implementation.	We changed the two RQs 4 and 5 to sharpen the focus of RQ4 on the eye-tracking parameters and the focus of RQ5 on the correlation of these parameters with salience:<br><br>(RQ 4) Do specific properties of the depicted objects in the HOPI (e.g., size, previously known species) correlate with the order objects are looked at?<br><br>(RQ 5) Do participants recall large objects or species previously known to them better?
7	With regard to the Methods section, I would kindly suggest clarifying the description provided in the following passage: "The study consisted of several stages. Firstly, the participants were shown the hidden object picture (Figure 2) for 30 s (for reasoning see description of sub-study 1), and we recorded their eye-movement with eye-tracking glasses. Secondly, the students received the free-listing recall task: 'What did you see in the picture? Write down a list of all objects in the picture you can remember.' Thirdly, after the free-listing recall task, students were presented with 10 images of plants and animals depicted in the hidden object picture and asked to identify them." In particular, it is not entirely clear how the set of 10 images (plants and animals) was selected. I would kindly suggest specifying the criteria used for this selection (e.g.,	Changed to: "Thirdly, after the free-listing recall task, students were presented with the images of the 10 most salient animal species and the 10 most salient plant species from study 1 (see Table 2) and asked to identify them."

	salience, size, position in the picture, or prior familiarity), as well as how many of these images correspond to plants versus animals.	
8	I would like to suggest some clarification regarding the conceptual distinction between attention, knowledge/understanding, and memory, as these constructs appear to overlap in parts of the introduction. I note that this point is also related to concerns raised by the reviewers, and I feel it could still be further strengthened in the current version.<br><br>In the introduction, attention is presented as one dimension of plant awareness, distinct from understanding and attitudes. However, in the subsequent discussion, particularly when referring to participants' responses to image prompts, it is not entirely clear how attention is being operationalised independently from recognition, prior knowledge, or recall processes. For instance, participants' ability to report or recall elements from an image may reflect not only attentional processes, but also memory and familiarity with the depicted objects.<br><br>This issue becomes more apparent in the formulation of the research questions. For example, RQ1 focuses on what participants "report" or recall from the image, which may primarily capture memory processes, while RQ3 explicitly refers to attention. More broadly, several of the research questions (RQ1, RQ2, RQ4–RQ7) appear to rely substantially on recall-based measures, which makes it less clear how each of them contributes to the validation of an instrument intended to assess attention. The distinction between these constructs, and how each research question maps onto them, could be further clarified. In particular, the statement that "research question 1 relates to the potential of the HOPI for exploring a person's attention towards plants" may benefit from additional justification, as recall-based measures do not necessarily provide a direct proxy for attention without a clear theoretical linkage. Overall, I would encourage the authors to more explicitly differentiate attention from related constructs such as recognition, prior knowledge, and memory, and to clarify how their methodological approach allows them to isolate or infer attentional processes.	We made clearer now that we operationalise "attention towards plants" in the plant awareness construct and within the frame of the paper presented not solely as pure "visual attention" but as a "selective attentional-memory process" (as also recommended by the reviewers – see comment 11). Furthermore, we emphasised that why we see, from an educational point of view, that rather the visual information that is passed to long-term memory (referring to recall) is key since it is the basis of learning processes.<br>Therefore, we consistently distinguished between "pure" *visual attention* (as reflected by eye-tracking) and selective attentional-memory processes (as reflected by the recall tasks).<br>In addition, all memory measures taken in the present study rely on the content of the presented images. Therefore, there is a connection between the two measures: visual attention (as selection of content from the images) is a necessary but not a sufficient precursor for the content that the participants recalled from the images. As you rightly infer, the latter also depends on participants' prior knowledge, their biases, etc.<br><br>Additionally, we added a conceptual figure (Figure 1) to make clear how attention and all its components are defined in this paper.

9	The discussion of salience is clear and well illustrated. However, I would suggest some caution regarding the interpretation of salience as a direct indicator of attention. While salience measures derived from free listing (e.g., frequency and order of recall) provide valuable insights into the prominence of items in collective memory, they are more directly linked to recall processes and shared knowledge. The relationship between salience and attentional processes is likely indirect and may depend on additional factors, such as prior familiarity or cognitive relevance. In this sense, the statement that salience allows the identification of elements that "dominate focused attention" may benefit from further qualification or theoretical justification. Alternatively, it could be helpful to clarify that this relationship is being assumed as a working hypothesis rather than a direct inference, or to acknowledge that the link between attention and salience requires empirical validation.	We changed the sentence to: "It enables the identification of the 'core' set of elements that dominate selective attentional-memory processes (…)" to align it also with the other changes aiming to clarify the operationalisation of attention in this paper.
10	The ideas presented in following section of the methodology ("Eye-tracking provided a straightforward measure of what participants paid visual attention to. However, the eye can be directed towards locations without the selection of relevant information from these measures...") offers a helpful and nuanced reflection on the relationship between visual attention (as measured by eye-tracking) and memory (as reflected in recall). In particular, the distinction between overt attention, covert attention, and the encoding of information into memory is very interesting. However, I would like to suggest that this conceptual clarification could be introduced earlier in the manuscript, particularly in the Introduction. At present, the theoretical relationship between attention and memory, and its implications for the choice of dependent variables, only becomes fully explicit at this stage of the Methods section. Bringing this discussion forward would help readers better understand the rationale underlying the study design from the outset.	We moved this and the following paragraph to the introduction and made some necessary changes to make clearer the operationalisation of attention in this paper as a selective attentional-memory processes. See exact changes in the track-changes version of the article.
11	Relatedly, it may be beneficial to further reflect on the consistent use of terminology throughout the manuscript. In several instances, "attention" appears to be used in ways that may also encompass processes of recognition, encoding, or recall. Clarifying whether the study is primarily addressing visual attention, memory-based recall, or a broader construct such as "information selection"	See answer to comments 8, 9, 10 above.

	or "attentional-memory processes" would strengthen conceptual precision and coherence.	
12	The reported result that eye-tracking parameters and object size did not significantly predict object salience (RQ4) is particularly interesting and has important implications for the theoretical framing of the study. This finding suggests that the salience of objects, as derived from recall-based measures, may not be directly associated with visual attention as captured by eye-tracking metrics. In other words, what participants ultimately recall, and report appears not to be strongly determined by where or how long they looked at specific elements in the image. This raises an important point for interpretation. As such, the relationship between attention (as measured through eye-tracking) and recall-based salience may be more indirect than initially assumed. In the discussion topic, I would therefore encourage the authors to further reflect on the implications of this finding for their conceptual framework, particularly regarding the use of recall-based measures as indicators of attention. Clarifying what dimension of cognition, the HOPI is most effectively capturing (visual attention, memory, or a broader notion of information selection) would strengthen the overall coherence of the manuscript.	See answer to comments 8, 9, 10 above.
